# Towards vitality: a longitudinal pilot study with a cognitive bias modification e-health intervention (VitalMe) to reduce fatigue in patients with chronic kidney disease

**DOI:** 10.1080/21642850.2025.2575779

**Published:** 2025-11-13

**Authors:** Jody A. Geerts, Christina Bode, Elske Salemink, Gozewijn D. Laverman, Femke Waanders, Nicole Oosterom, Peter M. ten Klooster, Marcel E. Pieterse

**Affiliations:** a Centre for eHealth & Well-being Research, Section Psychology, Health and Technology, University of Twente, Enschede, the Netherlands; b Department of Clinical Psychology, Utrecht University, Utrecht, the Netherlands; c Department of Internal Medicine, Division of Nephrology, ZGT, Almelo, the Netherlands; d Faculty of Electrical Engineering, Mathematics and Computer Science, University of Twente, Enschede, the Netherlands; e Department of Internal Medicine, Isala, Zwolle, the Netherlands

**Keywords:** Cognitive bias modification, attentional bias, self-identity bias, nephrology, fatigue

## Abstract

**Background:**

This longitudinal pilot trial investigated the effects of novel Cognitive Bias Modification (CBM) training targeting fatigue on cognitive biases, fatigue, vitality, and fatigue-related behaviour in people with chronic kidney disease (CKD).

**Methods:**

Thirty patients were alternately allocated to a week of CBM training with either attentional bias modification (ABM) or self-identity bias modification (SIBM), followed by a second week with the trainings combined. Twenty-two participants (12 pre-dialysis, 10 dialysis) completed the study, where cognitive biases and self-reported outcomes were measured at baseline, post-training, and follow-up. Possible interaction effects between CBM focus and disease stage were explored.

**Results:**

A significant effect of time was found on both cognitive biases; participants' attentional bias (Cohen's *d* = 0.88–0.99) and self-identity bias (Cohen's *d* = 1.16–1.28) were significantly more vitality oriented at post and follow-up compared to baseline. On the self-report outcomes, a small beneficial effect was found on vitality, but only for the ABM training.

**Conclusions:**

This is the first study to introduce CBM, which targets fatigue, to people with CKD. Despite the limitations in sample size and design, this study revealed strong effects on cognitive biases. It is recommended to replicate these findings in an adequately powered randomised controlled trial.

## 1. Introduction

Fatigue is reported as a frequent and important symptom by people with chronic kidney disease (CKD). The prevalence of fatigue in people with CKD ranges from 42% to 89% (Artom et al., 2014; Jhamb et al., 2008). Patients with end-stage CKD, who are usually dependent on haemodialysis, listed fatigue as their number one priority, independent of age, sex, or dialysis type (Tommel et al., 2023). For many patients, the experience of fatigue is linked to dialysis treatment. Next to a continuous fatigue that many patients experience, dialysis treatment is often followed by a decrease in energy levels, which can take hours to days to recover from, making some patients feel as if they are confined to a vicious circle of post-dialysis fatigue (Horigan & Barroso, 2016; Jacobson et al., 2019). Fatigue is associated with impaired quality of life and mental, social and societal functioning (Artom et al., 2014; Jacobson et al., 2019; Swain, 2000).

In patients undergoing haemodialysis, negative beliefs about fatigue, psychological distress, and fatigue-related behaviour, such as avoidance and all-or-nothing behaviour, influenced fatigue severity above and beyond clinical and demographic factors (Chilcot et al., 2016). Conventional health behaviour theories, as well as most existing interventions targeting the cognitive and behavioural factors of fatigue in patients with CKD (e.g. physical exercise and cognitive behavioural therapy), have focused on conscious and reflective processes to target fatigue (Artom et al., 2014; Sheeran et al., 2013). However, dual-process models, such as the reflective-impulsive model (RIM; (Strack & Deutsch, 2004)), propose two systems to process information and steer behaviour: the reflective system, which is conscious, rule-based, and deliberative, and the impulsive system, which is unconscious, associative, and instinctive (Houlihan, 2018; Sheeran et al., 2013).

One way unconscious processes can influence fatigue is through cognitive biases that can automatically get activated even if they counter conscious goals (Wiers & Wiers, 2017). Cognitive biases related to symptoms have already been found in multiple patient groups. For instance, people with multiple sclerosis and people with chronic fatigue syndrome (CFS) show an interpretational bias towards illness-related or negative information, added by an attentional bias in people with CFS (Hughes et al., 2016). Moreover, multiple cognitive biases (in attention, interpretation, and memory) have been found to play a role in pain (Pincus & Morley, 2001), a similarly multifactorial symptom as fatigue (Eccles & Davies, 2021).

The schema-enmeshment model (Pincus & Morley, 2001) explains how cognitive biases could influence symptoms: cognitive biases are thought to be the result of overlap between schemas of self, illness, and symptoms. During repeated experience of symptoms, the symptom schema can become enmeshed with the illness and self-schemas, resulting in cognitive biases (Pincus & Morley, 2001). Furthermore, to explain chronic fatigue development and how it is linked with avoidance behaviour, an associative learning model has been proposed (Lenaert et al., 2018). Irrespective of actual fatigue symptoms, the anticipation of fatigue on its own can trigger avoidance behaviour and fatigue symptoms. Cognitive biases are recognised as important factors for this associative learning process (Lenaert et al., 2018).

Cognitive bias modification (CBM) targets cognitive biases and aims to reverse the cognitive biases from negative to positive (MacLeod, 2012; Salemink et al., 2023) and improve clinical symptoms. Reviews and meta-analyses reveal that CBM has shown evidence for reducing anxiety and depressive symptoms (Fodor et al., 2020; Jones & Sharpe, 2017). Promising results have also been found for acute pain (Sharpe et al., 2012), chronic pain (Schoth et al., 2013; Sharpe et al., 2012) and fibromyalgia (Carleton et al., 2011). Furthermore, our research team has found promising results of similar CBM training on cognitive bias in people with breast cancer (Geerts, Pieterse, Sniehotta, Siemerink, Bode, under review; Geerts et al. under review). Thus, CBM has been found to be effective in reducing psychopathology and shows promise for fatigue and similar symptoms.

Previous research has also investigated possible moderators of CBM effects, such as symptom severity, and differences between targeted biases. The interaction between cognitive biases is receiving more attention with the upcoming combined cognitive bias hypothesis (CCBH) postulating that cognitive biases could interact and influence each other and together could have amplifying effects on symptoms (Leung et al., 2022; Van Ryckeghem et al., 2019), as reported in a meta-analysis (Martinelli et al., 2022) that compared multiple attentional and interpretational CBM studies and found positive effects on most symptoms. However, exceptions were also found. Therefore, more research is needed to investigate the interaction between cognitive biases and training effects.

Second, the rationale behind symptom severity as a moderator is that with more severe symptoms, cognitive biases would be more negative, giving more room for improvement and more to gain from training. Most meta-analyses did not find conclusive differences between clinical and non-clinical samples or only found differences between disease subgroups (e.g. general anxiety vs. PTSD), which could be explained by their characteristics or underlying mechanisms (Martinelli et al., 2022). Besides exceptions (e.g. Salemink et al., 2023), not many studies have investigated symptom severity as a moderator of CBM effect. As in CKD, fatigue is experienced to be connected to dialysis treatment, it is a potentially relevant moderator to investigate.

As fatigue is a prominent symptom in people with CKD and treatment options are limited (Yang et al., 2015), effective fatigue interventions are valuable additions to CKD care. As CBM consists of simple computer tasks that require little effort, both in terms of time and cognitive resources needed, it seems relatively acceptable for patients with a lack of energy due to severe fatigue. Indeed, CBM has been found to be a particularly promising treatment for people in stressful situations, possibly because of the implicit nature of the tasks and because it does not require as much active reflection as other interventions do (Bowler et al., 2012; Hallion & Ruscio, 2011). Additionally, CBM is easy to convert to an online setting (e.g. Wolters et al., 2021), making it a low-cost and 24/7 accessible option (Bowler et al., 2012; Wolbers et al., 2021). Although CBM could be a promising intervention for reducing fatigue symptoms, to our knowledge, its potential has not yet been studied for this purpose.

Therefore, we chose to develop and investigate two types of CBM training: attentional bias modification training (ABM), often investigated in previous studies, and novel self-identity bias modification training (SIBM), inspired by the schema-enmeshment model (Pincus & Morley, 2001). The aim of this pilot study was to explore the potential effectiveness of CBM training on cognitive biases and self-reported fatigue, vitality, and fatigue-related behaviour in people with CKD. For the cognitive biases, we expected to see a bias towards fatigue before the training to be converted into a bias towards vitality. We hypothesised a similarly beneficial effect on the self-report outcomes. Additionally, possible moderators, namely, CBM focus (ABM or SIBM) and disease stage (pre-dialysis or dialysis, were explored. Although the prevalence rates of fatigue are similar between pre-dialysis and dialysis patients, with prevalence rates of 78% to 81%, respectively (Almutary et al., 2013), patients undergoing dialysis often suffer from CKD for a longer time than patients in the pre-dialysis stage, and fatigue is often connected to dialysis. Therefore, it was hypothesised that patients undergoing dialysis would have significantly more fatigue bias at baseline than patients in the pre-dialysis stage and would respond significantly stronger to CBM training. Because not much is known about the separate and combined effects of ABM and SIBM training, no hypotheses were formulated for this moderator.

## 2. Materials and methods

### 2.1. Design

This study was a longitudinal non-randomised pilot trial with time as a within-subject factor. Cognitive biases (attentional and self-identity bias) were the primary outcomes, and self-report outcomes (fatigue, vitality, avoidance, and all-or-nothing behaviour) were secondary outcomes. The two moderators were between-subject factors with two levels: CBM focus (ABM or SIBM) and disease stage (pre-dialysis or dialysis). To investigate both the separate and combined training effects, participants were alternately allocated to the ABM or SIBM training in the first week and a combination of both training methods in the second week of training.

### 2.2. Participants

In an a priori power analysis (based on a repeated measures ANOVA, with 3 time-levels, 2 equal groups, 80% power, 5% alpha, and 0.50 correlation among repeated measures), it was determined that 24 participants were required to detect medium effect sizes (Cohen's *d* = 0.50–0.55). The participants were asked to participate by their nephrologist or nurse practitioner at Isala Hospital in Zwolle and the Hospital Group Twente (ZGT) in Hengelo and Almelo. Data collection occurred from January to April 2020. In total, 30 patients (*n*_Isala_ = 16, *n*_ZGT_ = 14) started the intervention study, of whom 6 patients stopped their participation because of (a combination of) health issues (*n* = 4), difficulties in laptop use and computer tasks (*n* = 3) and being busy (*n* = 1). Two participants did not indicate that they wanted to stop the study but only participated in the first measurements. Thus, 22 patients (mean age = 63, SD = 14, range: 26–84, for further demographic characteristics, see Supplemental file) completed the study.

#### 2.2.1. Inclusion and exclusion criteria

The inclusion criteria were (1) being diagnosed with CKD stage 4−5 (pre-dialysis) or 5D (dialysis), (2) being able to read and write Dutch, (3) having adequate sight for operating a computer, (4) having basic internet skills, and (5) having reported fatigue symptoms to their health practitioner. The exclusion criteria were (1) being scheduled for transplantation within 3 months and (2) having any somatic or psychiatric comorbidity that may impede adherence to the CBM or study protocols.

### 2.3. Materials

#### 2.3.1. Tasks

Attentional bias was measured with a modified visual probe task (VPT) (MacLeod et al., 1986) containing 100 trials (20 practice trials and 80 measurement trials). In each trial, pairs of fatigue and vitality stimuli were shown vertically for 1000 ms, followed by a dot. The participants reacted to the dot with the arrow keys (upwards and downwards, corresponding to above or below the middle of the screen). The stimuli (see Appendix A) and dot locations were randomised, and the dot appeared at the fatigue word at 50% of the trials.

Self-identity bias was measured with a modified Implicit Association Task (IAT) (Greenwald et al., 1998) containing 7 blocks (5 practice blocks and 2 test blocks) with a total of 200 trials. The participants first completed 3 practice blocks: (1) the target concepts (Fatigue vs. Vitality, 20 trials), (2) the attribute categories (Me vs. Other, 20 trials), and (3) the initial combination (Fatigue + Me vs. Vitality + Other, 20 trials). This was followed by the first test block (40 trials). Next, the category positions on the screen were switched. In the first part, fatigue was assigned to the left key, and vitality was assigned to the right. In the second part, fatigue appeared on the right, while Vitality appeared on the left. The participants practiced this reversed mapping with (4) the target concepts (vitality vs. fatigue, 20 trials) and (5) the new combination (vitality + Me vs. fatigue + other, 20 trials). Finally, they completed the second test block (40 trials). Based on the two test blocks, the intra-individual standardised difference in mean response latencies between fatigue congruent (Me + Fatigue) and incongruent (Me + Vitality) trials were calculated. The order of the stimuli was randomised, and the participants used the arrow keys (left and right) to categorise them. For both bias measures, positive scores indicate stronger fatigue bias, whereas negative scores indicate stronger vitality bias.

#### 2.3.2. Questionnaires

In the first measurement, participants were asked about demographic characteristics (i.e. gender, age, ethnicity, highest education, profession, marital and family status, and kidney disease stage). Subjective vitality, fatigue, avoidance, and all-or-nothing behaviour were measured with questionnaires. First, the Checklist Individual Strengths (CIS) (Vercoulen et al., 1994) contained 20 items reflecting fatigue severity, activity, concentration, and motivation. Each item (e.g. ‘Physically I feel exhausted’) was answered with a 7-point Likert scale (Yes, that is right — No, that is not right). Total scores (range = 20–140) indicate high fatigue and low motivation, concentration, and physical activity levels. A CIS score higher than 76 indicates problematic fatigue (De Vries, 2003). The CIS has shown excellent reliability, with a Gutman split-half reliability coefficient of 0.92 and a Cronbach's alpha of 0.90 (Schulte-van Maaren, 2014; Vercoulen et al., 1994). Second, vitality was measured with the validated Dutch vitality measure (Vita-16) (Strijk et al., 2015). The Vita-16 contained 16 items (e.g. ‘After dinner, I am still full of energy’) focusing on energy, motivation, and resilience. The items were answered on a 7-point Likert scale (1 = never, 7 = always). The answers were averaged, with high scores indicating high vitality. The Vita-16 has excellent reliability, with a Cronbach's alpha of 0.95.

Third, the behavioural subscales of the Cognitive and Behavioural Responses to Symptoms Questionnaire (CBSQ) (Knoop et al., 2012; Skerrett & Moss-Morris, 2006) were used to measure all-or-nothing and avoidance/resting behavioural responses to symptoms. The eight items on the avoidance/resting subscale (e.g. ‘When I experience symptoms, I rest’) and the five items on the all-or-nothing subscale (e.g. ‘I tend to overdo things and then rest up for a while’) were answered on a 5-point frequency scale (0 = never to 4 = all the time). Scores were added for each subscale, with higher scores indicating more all-or-nothing or avoidance behaviour (range = 0–20 for the all-or nothing subscale and 0–32 for the avoidance subscale). The behavioural subscales have good to acceptable internal consistency, with a Cronbach's alpha of 0.85 for the all-or-nothing subscale and 0.76 for the avoidance subscale (Loades et al., 2020).

### 2.4. Intervention

The Cognitive Bias Modification (CBM) training named VitalMe consists of 12 daily training sessions (6 sessions per week) for two weeks. The ABM training contained 5 rounds of 20 trials and used the VPT paradigm, but instead of the 50/50 allocation of the dot at the vitality and fatigue stimuli, the dot always appeared at the location previously occupied by the vitality word. In this way, the participants were guided to ignore the fatigue cues and instead direct their attention to the vitality cues. The SIBM training contained 3 rounds of 40 trials and used the IAT paradigm, but only used incongruent trials (Vitality + Me) instead of the combination of congruent and incongruent trials. In this way, participants were trained to pair vitality-related stimuli with self- and fatigue-related stimuli with other.

### 2.5. Procedure

As depicted in Table 1, this study took eight to nine weeks starting with a baseline phase (one or two weeks) with three measurements per week followed by a two-week treatment phase with a training session on six of the seven days, combined with one measurement per week. A five-week post-treatment phase was followed by three weekly measurements (one per week) and one final follow-up measurement two weeks later. After providing informed consent, the participants were sequentially assigned to one of four conditions: 1- or 2-week baseline and ABM or SIBM training in the first training week. In the second week, the participants received both training methods: first, the SIBM, then the ABM training.

**Table 1.** Schematic overview of the study design.

	Week 1 B*	Week 2 B	Week 3 T	Week 4 T	Week 5 F	Week 6 F	Week 7 F	Week 8 F	Week 9 F
Measurements	3/0^a^	3	1	1	1	1	1		1
Trainings			6: SIBM/ABM^b^	6: SIBM & ABM					

* B = baseline, T = training, F = follow-up.

^a^ Participants either received 1 or 2-week baseline.

^b^ In the first training week, participants either trained with the SIBM training or the ABM training. In the second training week, participants trained with both.

### 2.6. Data analyses

First, to test the within-group hypotheses, six linear mixed model (LMM) analyses were conducted with time (3 phases; baseline, post, follow-up) as a fixed factor and all outcome variables (attentional bias, self-identity bias, fatigue, vitality, avoidance, and all-or-nothing behaviour) as separate dependent variables. Second, to investigate the moderators, similar LMM analyses were conducted with time as a covariate and disease stage, CBM focus and their interactions with time added as fixed factors. Additionally, to test the hypothesis that the dialysis group would have more fatigue bias at baseline than the pre-dialysis group, a MANOVA was conducted with attentional bias and self-identity bias as the dependent variables and disease stage as between-subject factor.

Model assumptions were checked for all outcome variables. In the two bias variables, one residual outlier was identified. As these scores are the average of 6 baseline measures, they were considered unlikely to be caused by measurement error. Sensitivity analyses with time as a fixed factor did not show different results without outlier. However, sensitivity analyses with time, disease stage, and CBM focus as fixed factors did show different results without the outlier probably because of the small sample size. Therefore, it was decided to keep the outlier in the dataset for the training effect analyses but not for the moderator analyses.

### 2.7. Ethical statement

The Ethical Principles of Psychologists and Code of Conduct as set out by the British Association for Behavioural and Cognitive Psychotherapies and the British Psychological Society apply to this study. The Committee of Human Research [Commissie Mensgebonden Onderzoek, CMO] evaluated this study and decided that the Medical Research Involving Human Subjects Act [Wet medisch-wetenschappelijk onderzoek met mensen, WMO] did not apply (file number 2019-5816). The local ethical committees of the two participating hospitals and the faculty Behavioural, Management and Social Sciences of the university approved this study (file numbers 191020, 19−26, and 191193, respectively).

## 3. Results

### 3.1. Descriptives and correlations

The descriptive results and correlations at baseline can be found in Tables 2 and 3, respectively. The participants showed a neutral-to-slight fatigue bias at baseline (*M*_D_ = −0.04, *M*_VPT_ = 23.6), and the pre-dialysis and dialysis groups did not differ significantly on attentional bias (*p* = 0.294) or self-identity bias (*p* = 0.369). The D- and VPT-scores correlated significantly and strongly (*r* = 0.66, *p* = 0.004) in that a fatigue-oriented self-identity bias corresponded with a fatigue-oriented attentional bias. Surprisingly, the cognitive biases did not correlate with the self-report measures. Only vitality correlated marginally but moderately with attentional bias (*r* = −0.50, *p* = 0.07), indicating that more vitality bias corresponds with higher vitality scores. Furthermore, vitality correlated significantly and strongly with fatigue (*r* = −0.74, *p* < 0.001) but only marginally with avoidance (*r* = −0.40, *p* = 0.062) and not with all-or-nothing (*r* = −0.12, *p* = 0.581) behaviour. Fatigue correlated moderately to strongly with the behavioural measures in the expected direction (all-or-nothing: *r* = 0.45, *p* = 0.033; avoidance: *r* = 0.65, *p* < 0.001). Additionally, all-or-nothing behaviour and avoidance were significantly strongly intercorrelated (*r* = 0.71, *p* < 0.001).

**Table 2.** Descriptive statistics on all outcome variables at baseline.

	Total			Pre-dialysis			Dialysis		
	M	SD	*n*	M	SD	*n*	M	SD	*n*
Self-identity bias	−0.04	0.22	17	−0.24	0.28	7	−0.08	0.18	10
Attentional bias	23.56	70.33	17	45.59	107.37	7	8.14	21.57	10
Fatigue	86.74	23.45	23	86.5	18.82	11	86.96	27.88	12
Vitality	3.59	1.03	23	3.52	1.10	11	3.66	1.01	12
All-or-nothing	8.48	4.45	23	7.41	3.31	11	9.46	5.24	12
Avoidance	11.07	6.14	23	9.5	2.07	11	12.50	8.17	12

**Table 3.** Pearson correlations between all outcome variables at baseline.

Variable	*n*	1	2	3	4	5	6
1. Attentional bias	17/14^a^	–					
2. Self-identity bias	17/14	0.66**	–				
3. Fatigue	23	0.37	−0.07	–			
4. Vitality	23	−0.50	−0.27	−0.74**	–		
5. All-or-nothing	23	0.01	−0.42	0.45*	−0.12	–	
6. Avoidance	23	0.13	−0.21	0.65**	−0.40	0.71**	–

*Note:* *p < 0.05, **p < 0.001.

^a^ *n *= 17 for correlations between cognitive biases (attentional bias and self-identity bias), *n* = 14 for correlations between cognitive biases and self-reported outcomes.

### 3.2. Effect of CBM on bias and self-report measures

Time had a significant overall effect on attentional bias (*F*(2,40) = 6.01, *p* = 0.005), with significant post hoc differences between baseline and post (*p* < 0.002) and baseline and follow-up (*p* < 0.008). The separately calculated Cohen's d indicated that the effect was large (Cohen's *d* = 0.88–0.99). Participants showed a positive fatigue-oriented bias at baseline (*M* = 23.39, SE = 43.72) and a negative vitality-oriented bias at post (*M* = −20.19, SE = 43.89) and follow-up (*M* = −15.34, SE = 43.81). Similarly, time had a significant overall effect on self-identity bias (*F*(2,32) = 11.61, *p* < 0.001), with significant post hoc comparisons between baseline and post and baseline and follow-up (both *p* < 0.001). The separately calculated Cohen's d indicated that the effect was large (Cohen's *d* = 1.16–1.28). The participants showed a neutral bias at baseline (*M* = −0.04, SE = 0.18), which became more vitality oriented at post (*M* = −0.26, SE = 0.19) and follow-up (*M* = −0.28, SE = 0.18). The use of Sidak's HSD correction did not alter the significance of these findings. The analyses of self-reported outcomes did not yield any significant effects for time. Thus, while both cognitive biases changed strongly from neutral or fatigue bias to vitality bias, the self-report outcomes did not change over time.

### 3.3. Moderator effects

**Figure 1.**
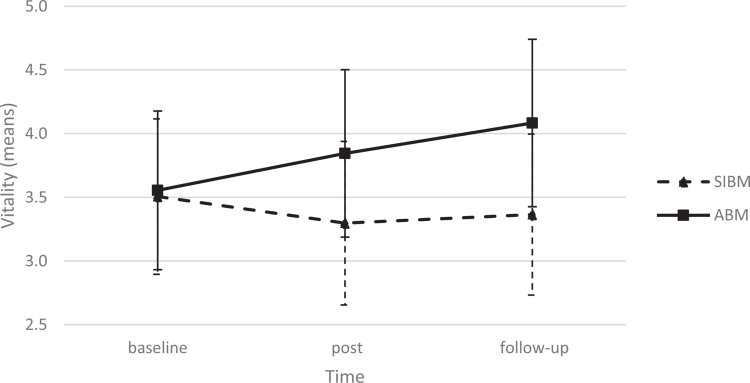
The interaction effect between time and CBM focus on vitality. Note: The means depicted are estimated marginal means. The error bars represent the 95% confidence interval. ABM = attentional bias modification; SIBM = self-identity bias modification.

In the analyses with the moderators added, only the above-described overall effect of time remained significant for attentional bias (*F*(1,36) = 6.06, *p* = 0.019) and self-identity bias (*F*(1,30) = 13.20, *p* < 0.001). Corresponding to the above results, no significant results were found for fatigue, avoidance, and all-or-nothing behaviour. For vitality, a marginal effect was found on the interaction between time and CBM focus (*p* = 0.053). To investigate this effect further, a separate LMM was conducted with CBM focus (ABM or SIBM) as a fixed factor and time as a covariate. In this analysis, the interaction effect was significant (*F*(1,30) = 5.29, *p* = 0.028), with an estimate of −0.33 (SE = 0.14, *t*(1,30) = 2.68, *p* = 0.028). As depicted in Figure 1, participants who received primarily the ABM training increased in their vitality scores comparing baseline (*M* = 3.56, SE = 0.30, *n* = 12) to post (*M* = 3.85, SE = 0.32, *n* = 8) and follow-up (*M* = 4.08, SE = −0.32, *n* = 8), while participants who received primarily the SIBM training remained stable in vitality scores (baseline: *M* = 3.51, SE = 0.30, *n* = 11, post: *M* = 3.30, SE = 0.32, *n* = 8, follow-up: *M* = 3.37, SE = 0.31, *n* = 9).

#### 3.3.1. Trajectory of change within the training weeks

**Figure 2.**
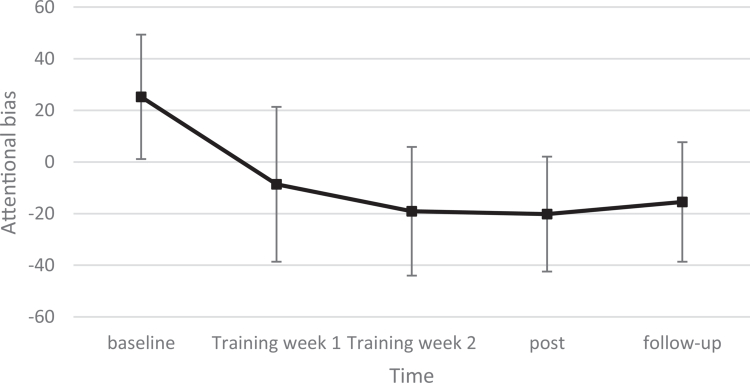
Trajectory of change in attentional bias. Note: This graph depicts estimated marginal means of attentional bias. The error bars represent the 95% confidence interval.

To explore the effects of the separate and combined trainings in training week 1 and 2, two LMMs were conducted with time as fixed factor with 5 timepoints (baseline, training week 1, training week 2, post and follow-up) and self-identity bias and attentional bias as dependent variables separately. Looking at the pairwise comparisons of the attentional bias analysis, all timepoints differed significantly from baseline (training week 1: *p* = 0.019, training week 2: *p* = 0.003, post: *p* = 0.002, follow-up: *p* = 0.006). As depicted in Figure 2, VPT-scores became more vitality-oriented during the training weeks (*M*_baseline_ = 25.23, *n* = 9, *M*_training week 1_ = −8.65, *n* = 10, *M*_training week 2_ = −19.07, *n* = 7), and remained relatively stable at post (*M* = −20.18, *n* = 7) and follow-up (*M* = −15.49, *n* = 9). This pattern remained in a sensitivity analysis without the outlier; however, the difference between training week 1 and baseline became marginal (*p* = 0.054).

**Figure 3.**
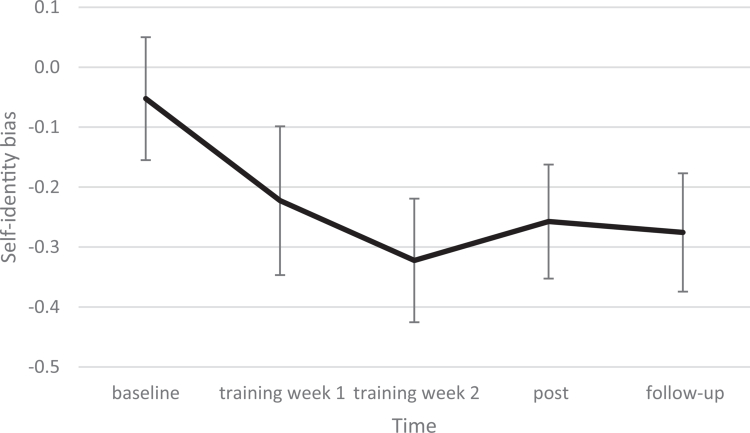
Trajectory of change in self-identity bias. Note: This graph depicts estimated marginal means of self-identity bias. Error bars represent the 95% confidence interval.

Similarly, on self-identity bias, all other timepoints differed significantly from baseline (training week 1: *p* = 0.021, training week 2: *p* < 0.001, post: *p* < 0.001, follow-up: *p* < 0.001). As depicted in Figure 3, the D-scores became more vitality oriented during the training weeks (*M*_baseline_ = −0.05, *n* = 8, *M*_training week 1_ = −0.22, *n* = 11, *M*_training week 2_ = −0.32, *n* = 4) and remained quite steady at post (*M*_post_ = −0.26, *n* = 9) and follow-up (*M*_follow-up_ = −0.28, *n* = 10). This pattern remained in a sensitivity analysis without the outlier; however, the difference between training week 1 and baseline became marginal (*p* = 0.050). For both biases, it seems that the largest training effect on attentional bias took place in the first week, while the second week did not appear to add much more. Regarding self-identity bias, a training effect seems visible in both training weeks, indicating that both the specified and the combined CBM had positive effects on self-identity bias.

## 4. Discussion

This study was the first to investigate CBM training countering fatigue in people with advanced CKD (pre-dialysis and dialysis stages). The expected effect of time was found on both cognitive biases: participants' attentional bias and self-identity bias became more vitality oriented at post-treatment and follow-up compared to baseline. No effects of time were found on symptoms or behaviours, except for a small positive training effect on vitality in the ABM condition. Notably, however, due to the lack of a control group, small sample size, and non-randomised allocation, these changes cannot be conclusively attributed to the training.

The finding that the training successfully modified the two biases, but not the self-reported outcomes, could possibly be explained by the baseline correlational findings. The cognitive biases correlated significantly with each other, but not or only marginally with the self-report measures. A correlation between cognitive biases and fatigue behaviour and symptoms was an important assumption for the potential effectiveness of the training. The findings of this study, challenge this reasoning and raise questions about whether these biases are mechanistically involved in symptom development and maintenance rather than being merely epiphenomenal. However, these results are in line with previous meta-analyses on CBM that found more robust evidence for cognitive bias change (with medium between-group effect sizes) but less consistent and smaller effects for actual symptom change by CBM training (Hallion & Ruscio, 2011; Martinelli et al., 2022), pointing to the option that cognitive biases may play a smaller, indirect or more gradual role in the development and maintenance of symptoms than presumed (Hallion & Ruscio, 2011; Martinelli et al., 2022).

Consistent with the marginal correlation found between attentional bias and vitality, a small effect of ABM training was found on vitality. This could be explained by a difference in information processing between the biases. Attentional bias is thought to operate at an early stage, while interpretation bias, memory bias, and presumably self-identity bias require more reflective thinking (Leung et al., 2022), possibly requiring stronger or longer training or more time for effects to translate to symptoms. This difference might also be reflected in our trajectory of change results: both trainings were successful in changing cognitive biases already within one week. The second week of training did not add much more of a training effect on attentional bias but did further foster self-identity bias. This finding, next to the strong correlation found between the two cognitive biases, is consistent with the combined cognitive bias hypothesis (Van Ryckeghem et al., 2019).

Moreover, as vitality is the counterpart of fatigue, it might be less embedded in the processes postulated by the schema-enmeshment model (Pincus & Morley, 2001) and the associative learning model (Lenaert et al., 2018) than fatigue and related behaviour, which could make it an easier outcome to be influenced by CBM training. Indeed, in the associative learning model (Lenaert et al., 2018), avoidance and fatigue are important end results, which could make them more difficult targets to change. It could be that the CBM training was not strong enough to influence these ingrained processes or that it takes longer for bias improvements to translate to changes in these outcomes. Although this study included follow-up measurements until one month after the first post-measurement, this might still not be long enough to detect delayed effects. For instance, chronic pain patients showed an effect of ABM on disability and anxiety sensitivity after 6 months (Sharpe et al., 2012).

Furthermore, it was hypothesised that disease stage would influence fatigue bias translating to a larger training effect in the dialysis group. These hypotheses were not met: the two patient groups did not significantly differ in their bias scores at baseline. This implies that people in both stages can benefit similarly from training. Indeed, both groups show extreme scores for fatigue and vitality: at baseline, 61% of the participants showed problematic fatigue (De Vries, 2003), and the average vitality score was lower than that of other people with chronic conditions (Strijk et al., 2015), which is in line with the fatigue prevalence rates found in patients with CKD (Almutary et al., 2013). These problematic scores highlight the urgency and importance of fatigue as a symptom for patients with CKD. Therefore, it is important to continue investigating interventions targeting fatigue in CKD patients.

To our knowledge, our research team is the first to develop CBM training targeting fatigue. ABM has been used and researched in many contexts but not previously in relation to fatigue. Self-identity bias in this form, to our knowledge, has not yet been studied in any context. Although the results suggest that the SIBM to be less effective than ABM, it remains important to investigate whether self-identity bias can influence symptoms and which mechanisms are most appropriate to train and measure it. To this end, we also adapted the VPT and IAT to measure the cognitive biases in the context of fatigue. Next to the question of content validity, all the measurements and training sessions used the same stimuli (see Appendix A), bringing to question the generalisability beyond the stimuli used. Moreover, the stimuli were taken from questionnaires measuring these constructs but were not further tested or matched for word length. Furthermore, although it is recommended to counterbalance the congruent and incongruent blocks in the IAT to control for an order effect (Schnabel et al., 2008), we always had the same order of blocks. Finally, in the VPT, error feedback and a second attempt were only provided in the practice trials. These methodological shortcomings in the design of the IAT and VPT should be addressed in future research (e.g. validation of stimuli and the use of different stimulus sets). Alternatively, other stimuli or categories with, for instance, more focus on the hospital setting, activities, or coping might induce more effects on fatigue-related behaviour.

Furthermore, an important limitation in the design of the current study is the lack of a control group. Because of this, it is unclear whether the found effects were solely caused by training. Therefore, this is an urgent recommendation for future research. Moreover, the low statistical power is the most obvious limitation of this study. This study already had a modest sample size, especially for the moderation analyses, and its power was further reduced by the unexplained missing data in the dataset (see Supplemental file). Initially, we planned to investigate the course of fatigue and fatigue bias in more detail over the weeks to investigate the variability of fatigue and the possible influence of dialysis. However, the details needed for this investigation were lost when we collapsed the data points. Similarly, we did not further explore the trajectory of change in the two training conditions because of the small sample size. Therefore, the variability of fatigue and the influence of dialysis on this, as well as the effects of separate and combined CBM trainings are recommended to be explored further. Additionally, the non-randomised allocation is a limitation. Although in the power analysis, only 2 groups were considered, this study incorporated many more groups, namely, the two moderators, CBM focus (ABM and SIBM) and disease stage (pre-dialysis and dialysis), as well as hospital (Isala and ZGT), baseline length (1 week or 2 weeks) and the measurement schedule. This, next to practical requirements, makes allocation complex. Allocation alternatives or at least randomised training allocation should be considered in future clinical studies.

Nevertheless, this is the first study to investigate an intervention targeting automatic processes (cognitive bias) to counter fatigue, a highly invasive symptom for people with CKD. Despite the limitations of this pilot study, it is encouraging that consistent and lasting beneficial effects of time are found on cognitive biases. Furthermore, the explorations in this study provide an indication of a promising effect of attentional bias modification training on self-reported vitality, as well as some confirmation of the combined cognitive bias hypothesis. Next to the already mentioned suggestions for future research, replication of this result, either with the same or other patient populations with fatigue and with other CBM paradigms, is recommended.

## Supplementary Material

Supplementary material Supplementary information.

## Data Availability

The data that support the findings of this study are available on request from the corresponding author. The data are not publicly available due to privacy or ethical restrictions.

## Appendix A Stimuli

### Stimuli VPT.

**Table:**

Fatigue words			Vitality words	
Dutch	English	*N*	Dutch	English
Moe	Tired	1	Actief	Active
Zwaar	Heavy	2	Fit	Fit
Uitgeput	Exhausted	3	Doen	Do
Moeite	Effort	4	Bezig	Busy
Sloom	Slow	5	Concentratie	Concentration
Slap	Weak	6	Uitgerust	Rested
Machteloos	Powerless	7	Aandacht	Attention
Vermoeidheid	Fatigue	8	Sterk	Strong
Vermoeid	Fatigued	9	Ondernemend	Venturous
Moeheid	Tiredness	10	Plannen	Plan
Problemen	Problems	11	Meedoen	Join in
Belemmerend	Obstructive	12	Zin hebben	Looking forward
Hinderen	Hinder	13	Gemotiveerd	Motivated
Beperkt	Limited	14	Bewegen	Moving
Loom	Lethargic	15	Bereiken	Reach
Afgemat	Overworn	16	Inzet	Commitment
Doodop	Knackered	17	Toewijding	Dedication
Lamlendig	Lame	18	Oplettend	Observant
Afgepeigerd	Outworn	19	Vitaal	Vital
Gevloerd	Floored	20	Energiek	Energetic
Flauw	Faint	21	Levendig	Lifely
		22	Druk	Load
		23	Dynamisch	Dynamic
		24	Gezond	Healthy
		25	Levenslustig	Full of life

### Stimuli IAT.

**Table:**

Target concepts				
	Dutch	English	Dutch	English
*N*	Vitaal	Vital	Moe	Tired
1	Energiek	Energetic	Uitgeput	Exhausted
2	Levenslustig	Full of life	Slap	Weak
3	Fit	Fit	Lusteloos	Apathetic
4	Wakker	Awake	Traag	Slow
5	Actief	Active	Duf	Dull
6	Alert	Alert	Slaperig	Sleepy
7	Krachtig	Powerful	Krachteloos	Powerless
8	Sterk	Strong	Vermoeid	Fatigued
9	Snel	Quick	Futloos	Lifeless
10	Vitaal	Vital	Moe	Tired

**Table:**

Attributes				
	Dutch	English	Dutch	English
N	Ik	I	Ander	Other
1	Zelf	Self	Hen	Them
2	Mijn	Mine	Ander	Other
3	Mij	Me	Hun	Their
4	Ik	I	Anderen	Others
5	Mezelf	Myself	Jullie	You
