# Supplementary material for: Towards vitality: a longitudinal pilot study with a cognitive bias modification e-health intervention (VitalMe) to reduce fatigue in patients with chronic kidney disease
Source: Health Psychol Behav Med. 2025 Nov 13;13(1):2575779. doi: 10.1080/21642850.2025.2575779 (PMC12616661; doi:10.1080/21642850.2025.2575779)
Supplement: Supplementary material — Supplementary information. [file RHPB_A_2575779_SM7277.docx]

**Supplemental File**

To describe the methodology as detailed as possible for replication goals, the reader can find further details on the study procedure, measurements, and data analysis in this attachment.

**Method**

**Table 1**

*Demographic Characteristics at Baseline*

| Baseline characteristic | N | % |
| --- | --- | --- |
| Dialysis | 10 | 45% |
| Female | 11 | 50% |
| White | 22 | 100% |
| Highest education level |  |  |
| Primary | 1 | 4.5% |
| Secondary or vocational | 18 | 82% |
| Higher | 3 | 14% |
| Employment |  |  |
| Unemployed (retired / disabled) | 14 | 64% |
| Employed | 8 | 36% |
| Marital status |  |  |
| Married / partnered | 12 | 55% |
| Divorced / widowed | 7 | 32% |
| Single | 3 | 14% |

*Note.* N = 22.

**Tasks**

The tasks used for this study were designed with Gorilla Experiment Builder (www.gorilla.sc). The task examples on this platform were used and adapted to fit our purposes. The VPT aims to measure attentional bias by shortly and simultaneously presenting pairs of fatigue related and vitality related stimuli, after which participants have to react to a probe that appears on the same location as one of the stimuli (Todd et al., 2018). People that have an attentional bias towards fatigue typically shift attention to the stimuli representing that concept, resulting in shorter reaction times when the probe appears at the same location compared to when it appears at the other word’s location (MacLeod et al., 1986). The IAT aims to measure automatically activated associations between a two-sided target (me vs. other) and a two-sided attribute (fatigue vs. vitality) with series of sorting tasks requiring fast responses (Schnabel et al., 2008). By comparing reaction times, the strength of associations between the different combinations can be calculated (Schnabel et al., 2008), in this case a self-identity bias towards fatigue or vitality.

Because of the longitudinal design of the current study, participants only received the practice blocks in the first week (3 measurements) of the study. After that, they received an adapted IAT starting immediately with a measurement block of the congruent combinations (40 trials), followed by a practice block of the switch (20 trials) and then a second measurement block (40 trials). Similarly, in the VPT, the participants only received a measurement block (80 trials).

The keys used for the tasks were adapted from the original IAT and VPT tasks that instruct to press the ‘E’ key with the left hand and ‘I’ key with the right hand corresponding with the left and right categories or the probe, respectively. During the design process of the current study, the keys were changed to the arrow keys (left and right for the IAT, up and down for the VPT) so that the tasks could be done with one hand during haemodialysis (as one arm is connected to the dialysis machine and should move as little as possible).

**Questionnaires**

To reduce workload on participants, the measurements contained either the multi-item questionnaire or a single-item questionnaire measuring only vitality and fatigue with two Visual Analogue Scales (VAS, e.g., ‘How tired / vital do you feel at this moment?’). The multi-item questionnaire was asked once a week at baseline (measurement 1 and 6), at post (measurement 9) and two times at follow-up (measurement 11 and 12).

**Procedure**

Participants were encouraged to do the measurements and trainings at home or during dialysis. Participants could choose a measurements schedule to best fit their own preferences and (dialysis) schedule (e.g., measurements on Monday, Wednesday and Thursday vs. Tuesday, Thursday, and Friday). The assessment and training tasks were administered via a combination of the Qualtrics survey platform (www.qualtrics.com) and Gorilla Experiment Builder (www.gorilla.sc). Due to the different features in the two platforms, it was decided to contact, instruct, and survey the participants via Qualtrics, and to direct the participants to Gorilla for the assessment- or training tasks (see Table 2). In total, a measurement or training took 5 to 15 minutes. E-mail reminders were sent to participants if they had not responded before a certain time (the same evening, the next day or a couple of days later).

**Table 2**

*Measurement Schedule and Recorded Data*

| Week | Phase | Measurement | Multi-item questionnaire | Observations | n Completed Qualtrics questionnaires | n Completed Computer tasks |
| --- | --- | --- | --- | --- | --- | --- |
| 1 | Baseline | 1 | X | 13 | 12 | 8 |
| 1 | Baseline | 2 |  | 12 | 12 | 7 |
| 1 | Baseline | 3 |  | 12 | 12 | 5 |
| 2 | Baseline | 4 |  | 22 | 20 | 13 |
| 2 | Baseline | 5 |  | 22 | 22 | 12 |
| 2 | Baseline | 6 | X | 21 | 19 | 12 |
| 3 | Training | 7 |  | 19 | 19 | 13 |
| 4 | Training | 8 |  | 22 | 22 | 17 |
| 5 | Post | 9 | X | 21 | 16 | 19 |
| 6 | Follow-up | 10 |  | 21 | 17 | 17 |
| 7 | Follow-up | 11 | X | 17 | 15 | 15 |
| 9 | Follow-up | 12 | X | 18 | 15 | 16 |

In total, 26 (10.8%) completely missing data points were observed. Of the 220 remaining available data points, an additional 19 (8.6%) missing data points were observed in the data obtained via Qualtrics, and 66 (30%) missing data points were observed in the data obtained via Gorilla.sc. Twenty-two (25.8%) of these could be explained by technical reasons registered during monitoring (e.g., participants stopped early, or something went wrong with the Gorilla link). Sixty-three datapoints (28.6%) were missing for unknown reasons.

**Data analysis**

***Data preparation***

For the attentional bias from the VPT measurements, mistakes (447 trials, 3%), and outliers (reaction times below 200 msec (74 trials, 0.5%) and above 2000 msec (126 trials, 0.9%), total 200 trials, 1.5%) were filtered out (e.g., Koster et al., 2004) as well as the practice trials. The final VPT scores were calculated by subtracting the mean reaction times on the fatigue trials (i.e., the trials were the probe appeared at the fatigue stimuli) from the mean reaction times on the vitality trials. For the self-identity bias, consistent with the conventional scoring algorithm (Greenwald et al., 2003), first the IAT data was cleaned by removing mistakes (1051 trials, 6%), outliers (reaction times below 400 msec (22 trials, 0.1%) and above 10.000 msec (58 trials, 0.3%), as well as the practice trials. With the remaining trials, the D-score was calculated by subtracting the mean reaction times on the congruent trials from the mean reaction times on the incongruent trials and by subdividing this by the overall standard deviation on all reaction times per participant (Greenwald et al., 2003).

Originally, it was planned to separately analyse all 12 data points to give a more detailed picture of the course of fatigue symptoms, as well as the changes due to training. The variable baseline (1- or 2-weeks) was also added for the exploration of the course of fatigue symptoms. However, because of the relatively large number of missing datapoints in the dataset (see Supplemental Material), it was decided to collapse repeated measurements in each phase. Collapsing multiple measurements across each phase was assumed to adequately deal with the missing measurements within each phase and improve the reliability of score estimates per phase. This means that for each dependent variable mean scores were calculated for each participant per phase (i.e., the mean of all baseline measurements, one post measurement (measurement 9), and the mean of the three measurements of the follow-up phase).

With the baseline data, Pearson correlations between all outcome variables were analysed. To explore the effects of the separate and combined trainings, the trajectory of change during the training weeks was investigated by adding the two measurements during training to the time-variable giving it 5 levels: baseline, training week 1, training week 2, post, and follow-up. Two LMM analyses were conducted with this time variable as fixed factor and attentional bias and self-identity bias as separate dependent variables.

***Reasons for LMM analyses***

LMM can inherently deal with randomly missing data as well as the multilevel nature of the data with measurements (level 1) nested within individuals (level 2) over time (Twisk, 2013). Restricted maximum likelihood estimation was used and the covariance structure for the repeated measurements was set to compound symmetry as this structure tended to show a better fit than an autoregressive AR(1) or unstructured matrix across the models of the different outcomes of interest with time as the only fixed effect according to the Akaike and Bayesian information criteria.

**Results**

**Training usage**

Of the 23 participants that did the training sessions, seven (30%) completed all 12 training sessions, five (22%) fulfilled all but one, and six participants (26%) missed two or three sessions. In the first week, 11 (48%) participants had done all the training sessions, six (26%) had done all but one, and four (17%) had done all but two. In the second week, 10 (43%) had done all training sessions, seven (30%) had done all but one training session, and five (22%) had done four or less training sessions. Only one participant missed the whole second week of training sessions (but did 4 sessions in week 1). Besides that, the lowest number of training sessions within a training week was 2 sessions (*n* = 1 in week 1, *n* = 2 in week 2). However, these participants did 5 sessions in the other week.
